# Machine learning–based classification of spatially resolved diffuse reflectance and autofluorescence spectra acquired on human skin for actinic keratoses and skin carcinoma diagnostics aid

**DOI:** 10.1117/1.JBO.30.3.035001

**Published:** 2025-03-04

**Authors:** Valentin Kupriyanov, Walter Blondel, Christian Daul, Martin Hohmann, Grégoire Khairallah, Yury Kistenev, Marine Amouroux

**Affiliations:** a Université de Lorraine, CNRS, CRAN UMR, Vandoeuvre-Lès-Nancy, France; b Tomsk State University, Laboratory of Laser Molecular Imaging and Machine Learning, Tomsk, Russia; c Friedrich-Alexander-Universität Erlangen-Nürnberg (FAU), Institute of Photonic Technologies (LPT), Erlangen, Germany; d Metz-Thionville Regional Hospital, Department of Plastic, Aesthetic and Reconstructive Surgery, Ars-Laquenexy, France

**Keywords:** skin cancer, optical biopsy, multimodal spectroscopic methods, diffuse reflectance spectroscopy, autofluorescence spectroscopy, machine learning

## Abstract

**Significance:**

The incidence of keratinocyte carcinomas (KCs) is increasing every year, making the task of developing new methods for KC early diagnosis of utmost medical and economical importance.

**Aim:**

We aim to evaluate the KC diagnostic aid performance of an optical spectroscopy device associated with a machine-learning classification method.

**Approach:**

We present the classification performance of autofluorescence and diffuse reflectance optical spectra obtained *in vivo* from 131 patients on four histological classes: basal cell carcinoma (BCC), squamous cell carcinoma (SCC), actinic keratosis (AK), and healthy (H) skin. Classification accuracies obtained by support vector machine, discriminant analysis, and multilayer perceptron in binary- and multi-class modes were compared to define the best classification pipeline.

**Results:**

The accuracy of binary classification tests was >80% to discriminate BCC or SCC from H. For AK versus other classes, the classification achieved a 65% to 75% accuracy. In multiclass (three or four classes) classification modes, accuracy reached 57%. Fusion of decisions increased classification accuracies (up to 10 percentage point-increase), proving the interest of multimodal spectroscopy compared with a single modality.

**Conclusions:**

Such levels of classification accuracy are promising as they are comparable to those obtained by general practitioners in KC screening.

## 1 Introduction

In 2022, keratinocyte carcinoma (KC) was the fifth most common cancer type in the world.^1^ Basal cell carcinoma (BCC) and squamous cell carcinoma (SCC) are the most common types of KC, accounting for 70% and 25% of cases, respectively. Metastasis occurs in only 1 out of 14,000,000 cases for BCC and in <10% for SCC,^2^ inducing an overall 90% 5-year survival rate. The age-standardized incidence rate of KC increased from 54.08/100,000 in 1990 to 79.10/100,000 in 2019 (+1.78% per year), and the number of reported cases is increasing every year.^3^^–^^5^

This increasing incidence entails an important medico-economic burden. Indeed, the standard procedure for suspicious skin lesion diagnosis includes surgical biopsy and histopathological analysis of the removed tissue samples.^6^^,^^7^ The ratio of biopsies to treatment increased from 1.1 in 1993 to 2.1 in 2016.^8^ In addition, biopsy is an invasive, long, and expensive procedure, which induces stress and scars and has low diagnostic accuracy as 20% of the biopsies only reveal skin cancers and two-thirds of the carcinomas are not diagnosed.^8^ Intraoperative delineation of healthy resection margins must also be optimized because the cancer is not completely removed in one quarter of the cases. Considering the number of patients with KC, this significantly increases the total economic burden.^9^ These statistics highlight the significant need for methods to aid in the diagnosis of KC.

At the same time, optical methods for skin examination may provide diagnostic information within a few minutes (versus a few days for histopathology) and can be used *in vivo*^6^^,^^10^^,^^11^ (thus avoiding invasive procedures). These advantages make “optical biopsy” methods promising diagnostic real-time assistance tools for clinical practice. The diagnostic potential of “optical biopsy” methods lies in their sensitivity to optical properties modifications of skin at the tissue, cellular, and subcellular scales, which are directly linked to pathological morphological and metabolic modifications during cancerous lesion development.

For more than two decades now, different optical spectro-imaging methods have been investigated for human skin cancer *in vivo* detection and/or diagnosis,^10^^,^^12^^,^^13^ among which: (i) diffuse reflectance spectroscopy (DRS)^14^^–^^16^ sensitive to the presence of absorbing elements (e.g., chromophores, melanin, and blood hemoglobin) in the skin and to morphological changes due to cancerous lesions, which impact the elastic scattering of light (disappearance of the layered structure, disorientation of cells, modification of size nuclei), (ii) autofluorescence spectroscopy (AFS)^17^^–^^19^ and fluorescence lifetime imaging microscopy^20^ sensitive to microenvironments and cellular metabolism altered during tumor progression, namely, through endogenous fluorophores present in dermis (elastin, collagen) and in epidermis (flavins, nicotinamide adenine dinucleotide), (iii) Raman spectroscopy (RS),^21^^–^^25^ which makes it possible to extract chemical and crystalline, structural and metabolic information, which is linked to the vibrational state (inelastic diffusion) of the molecules constituting the proteins and lipids of the skin, (iv) hyperspectral imaging,^26^^–^^28^ which combines high spatial and spectral resolutions, thus providing skin pictures with enhanced spectral qualities for analyzing absorption and scattering features, (v) reflectance confocal microscopy^29^ and optical coherence tomography (OCT),^30^^–^^32^ which is an in-depth imaging modality being sensitive to the refractive index variations among the various constituents in skin (organelles, cells, fibers, extracellular matrix). OCT provides vertically oriented cross-sectional images at the cellular level (for line-field OCT) and down to the superficial dermis. However, there is no single method that provides information about changes in all skin optical properties. This issue can be partially solved using multimodal methods, which involve the simultaneous use of several measurement methods in a single study.^33^^,^^34^ For instance, among the most studied multimodal approaches tested in clinical practice for *in vivo* skin cancer diagnosis, DRS and AFS with one and several excitations were combined,^35^^–^^37^ and RS demonstrated its further contribution to colocalized measurements in DRS and AFS^34^^,^^38^^,^^39^ or OCT.^40^ Furthermore, regarding tissue point spectroscopy methods, spatially resolved (SR) configurations using a point light source and several detectors located at different source-to-detector separations (SDSs) showed major impacts in the diagnostic characterization of pre-cancerous and/or cancerous skin lesions *in vivo*^14^^,^^41^^–^^45^ as well as in several other organs such as cervix, colon, bladder, and esophagus.^46^^–^^49^ Indeed, the detection of multiply scattered photons at short/long SDSs is related to their shallow/high depth trajectories in tissues, which is of particular interest for probing tissue pathology-related modifications such as in skin carcinogenesis, arising and developing throughout the epidermis layer then progressing toward the dermis and deeper for invasive stages.

Our group developed and patented a multimodal spectroscopy medical device^50^^,^^51^ combining SR diffuse reflectance (DR) and multiply-excited AF spectroscopies, which was implemented first in preclinical studies on mice skin cancer models *in vivo*.^44^^,^^52^ Recently, our group also published a unique and fully open database of more than 26,000 DR and AF spectra, acquired *in vivo* on 131 patients in clinics, featuring BCC, SCC, and actinic keratosis (AK).^53^ Consequently, processing and analyzing such complex and multi-dimensional datasets involve major challenges. With the much larger number of variables (i.e., numerous features compared with the number of individuals and the high levels of intra- and inter-patient variabilities), advanced methods of machine learning (ML) and statistics are necessary to tackle the curse of dimensionality, to mitigate overfitting and to extract relevant discriminant features. A solution for these challenges is required to reach an efficient differentiation among different classes of skin pathologies.

Various supervised classification approaches such as support vector machine (SVM),^54^^–^^56^ logistic regression,^15^ discriminant analysis (DA),^35^^,^^52^ and different variations of artificial neural network (ANN)^14^^,^^18^ have been implemented to identify different types of skin cancers based on AF and DR spectra. However, the performance of classifiers can significantly vary depending on the parameter adjustments and the data to be analyzed; therefore, it is necessary to compare the performance of several classifiers and perform hyperparameter optimization for each of them to improve results. Another important aspect is the effective use of multimodal data. For this purpose, it is possible to exploit data fusion methods, which have been successfully tested in preclinical studies.^55^^,^^56^

The originality of the present contribution is to provide an ML model performing supervised binary- and multi-class classifications of BCC and SCC, precancerous lesions (AK), and healthy human skin based on a unique set of spatially resolved DR and AF spectra. It is also important to note that, in addition to the diagnostic type of carcinoma (BCC or SCC), the dataset also contained information on whether the carcinomas were invasive or non-invasive (CI and CNI, respectively). Information about the invasiveness of the lesion may allow clinical specialists to determine the risk of local metastasis and, as a consequence, to prescribe more appropriate treatment for the patient. Besides the preprocessing, feature extraction/selection, and classification stages, different data fusion strategies are proposed to make the best use of the different modalities of DRS and multiply excited AFS. The study also investigates the effectiveness of the use of various spectra filtering and normalization methods.

The paper is organized as follows. Section 2 describes (i) the experimental protocol conducted in clinics, which includes the instrumentation and the measurement procedure, (ii) the spectroscopic and clinical data set obtained, and (iii) the data pre-processing and processing pipeline developed. Section 3 presents and discusses the results of skin lesion binary and multi-class classification before and after data fusion as well as in comparison with existing studies on AF and DR spectroscopy for *in vivo* diagnosis of human skin cancer.

## 2 Materials and Methods

### 2.1 Experimental Protocol Conducted in Clinics

The clinical study was conducted by a plastic surgeon at the Metz-Thionville Regional Hospital (France) and approved by both the French National Drug Agency (ANSM) and an ethical committee (CPP Est III) under the reference DMDPT-RIAL/MM/2016-A00608-43. Between 2017 and 2021, the clinical trial named “SpectroLive” included 131 patients with one or more lesions supposed to be skin carcinoma. A brief description of the experimental design is provided hereafter, but detailed materials and methods information can be found in Ref. 53.

#### 2.1.1 Instrumentation and measurement procedure

**Fig. 1:**
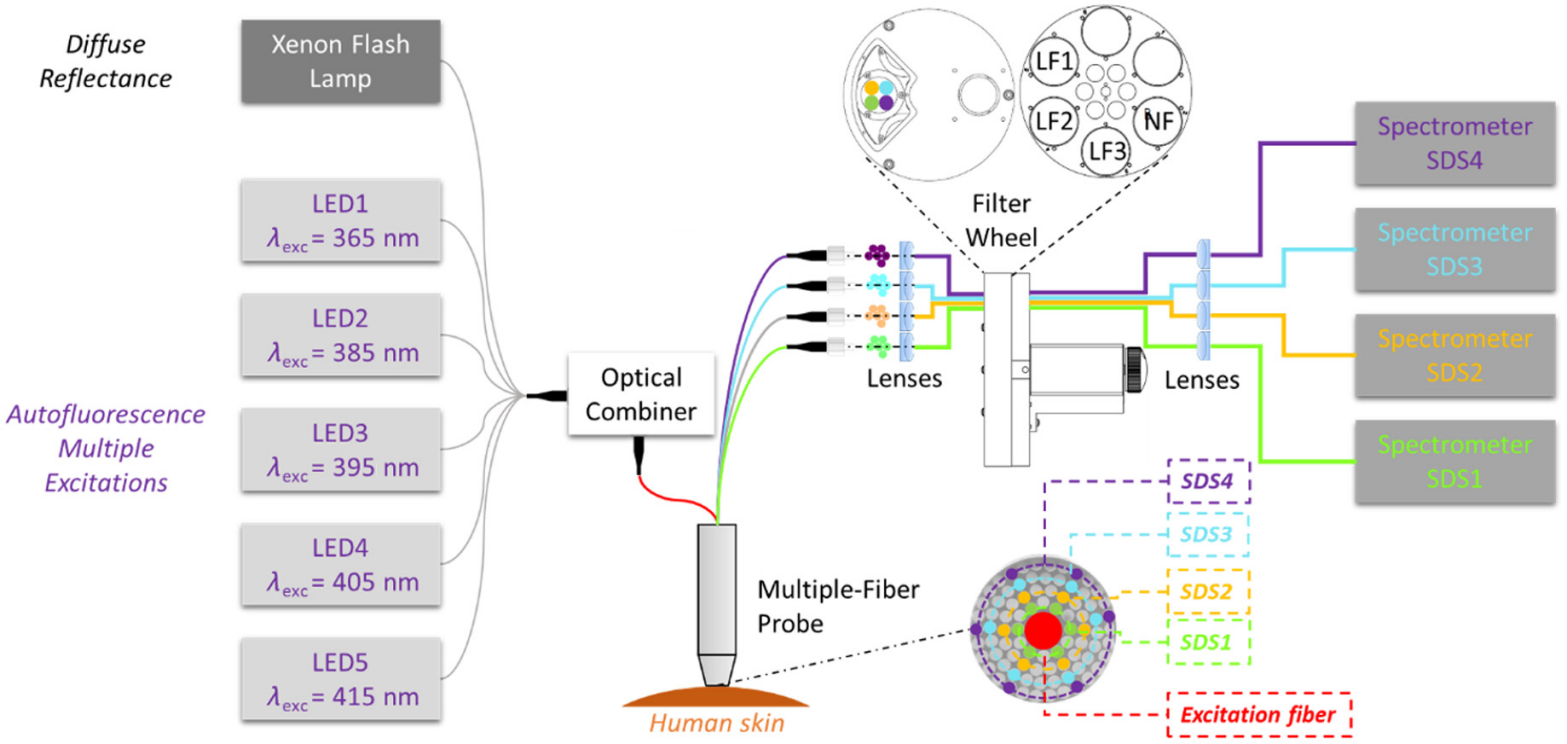
Simplified schematic representation of the SpectroLive medical device, including a Xenon lamp wide-band source for DR measurements and five LED narrow-band sources for AF excitations, the multiple-fiber optics probe featuring four source-detector separations (SDS1-4), a filter wheel equipped with long-pass emission filters for AF measurements and four spectrometers. Adapted with permission from Blondel et al.^50^

The instrumentation used in this clinical study was the patented spatially resolved DR and multiply excited AF spectroscopy device SpectroLive.^51^ A simplified scheme of the Spectrolive device is provided in Fig. 1 (detailed technical and metrological information can be found in Ref. 50).

The device is equipped with five high-power fiber-coupled light emitting-diode (LED) modules (Mightex^®^) LED1-5 in combination with in-line band-pass filters to provide narrow-band excitation peaks between 365 and 415 nm (see details in Table 1). A Xenon flash lamp from Hamamatsu (Massy, France) was used as a wide-band light source to obtain diffuse reflectance spectra (DRS) (see again Table 1). It has to be noticed that a Lambertian surface spectral on target (SRS-99-010, Labsphere^®^, Poynton, Great Britain) with a spectrally flat response in the wavelength range of acquisition was used to obtain the reference signal for DR spectra measurements.

**Table 1:** Main light source and detection characteristics of the SpectroLive device.

Modality	Source wavelength (nm)	Integration time (s)	Acquisition spectral range (nm)	Source-detector separation (μm)
DRS	Range: 350 to 800	0.05	340 to 785	${\mathrm{SDS}}_{1}=400$
Multi-excitation AF spectroscopy	Peak (FWHM^a^):	1	Cropped range^b^	${\mathrm{SDS}}_{2}=600$
	${\mathrm{AF}}_{1}=365$ (4)		420 to 785	${\mathrm{SDS}}_{3}=800$
	${\mathrm{AF}}_{2}=385$ (9)		420 to 785	${\mathrm{SDS}}_{4}=1000$
	${\mathrm{AF}}_{3}=395$ (7)		420 to 785	
	${\mathrm{AF}}_{4}=400$ (9)		440 to 785	
	${\mathrm{AF}}_{5}=415$ (10)		450 to 785	

a FWHM, full width at half maximum.

b Cropping was performed to limit the influence of remaining excitation light passing through the long-pass filter.

The optical probe has a measuring area diameter of 3.5 mm and contains one central 600-μm-diameter fiber for illuminating the skin tissue and four groups of six 200-μm-diameter fibers each, corresponding to four source-detector separations (SDS1-4) from 400 to 1000  μm between the centers of the light emitting and collecting fibers. The use of multiple SDSs allows us to vary the proportion of photons detected from deeper layers of the skin. The light collected by each group of six fibers is transmitted to four spectrometers (Mightex^®^, Pleasanton, CA, United States), respectively. On the way of the light, a filter wheel allows, before the spectrometers, for selecting appropriate long pass AF emission filters providing high-blocking (optical density >6) of the AF excitation peaks. The main characteristics of the device are presented in Table 1.

A “single sequence” measurement lasts 6 s and consists of the acquisition of 24 spectra, including 20 AF spectra (captured for five excitation peaks at four SDSs), and four DR spectra (one source signal and measurement at four SDSs). In practice, this single measurement was repeated three times for each tissue point (without moving the probe) to provide “single point” measurements with an increased signal-to-noise ratio or reduced influence of random outliers in the resulting spectra (see Sec. 2.2.2). The integration time, chosen to have an optimal compromise between signal-to-noise ratio and clinical ergonomics, was one second for each of the autofluorescence (AF) spectra and 50 ms for the DRS.^50^ In addition, dark noise was measured during each measurement to account for the influence of thermally generated electrons.

The SpectroLive device is essentially bimodal in that it provides both AF and DR modalities; however, it features five sources of AF excitation. As a consequence, each of the sources of excitation of AF will be considered an independent modality for data fusion steps in our classification pipeline to simplify the description of the methods used and the results obtained.

#### 2.1.2 Clinical and spectroscopic dataset

Extended information about the dataset can be found in Ref. 53. The primary results of the analysis of this dataset have been presented in Ref. 57. Briefly, the total number of lesions was 218. The age of all patients, their gender, their skin phototype, and the anatomical sites where the lesions were located are presented in Table 2.

**Table 2:** Distribution (%) of the 131 patients per gender, age class, phototype, and anatomical sites on which lesions developed (sometimes several lesions per patient).

Patient parameters (218 lesions for 131 patients)		Distribution (%)
Gender	Male	69
	Female	31
Age	30 to 50	2
	50 to 60	6
	60 to 70	18
	70 to 80	27
	80 to 90	37
	90 to 100	11
Phototype	I	13
	II	65
	III	20
	IV	2
Anatomical site	Forehead, vertex, ear, temple, eyelid	55
	Cheek, nasolabial fold	23
	Neck, nape, shoulders	10
	Back	4
	Arm, hand	4
	Legs	4

**Fig. 2:**
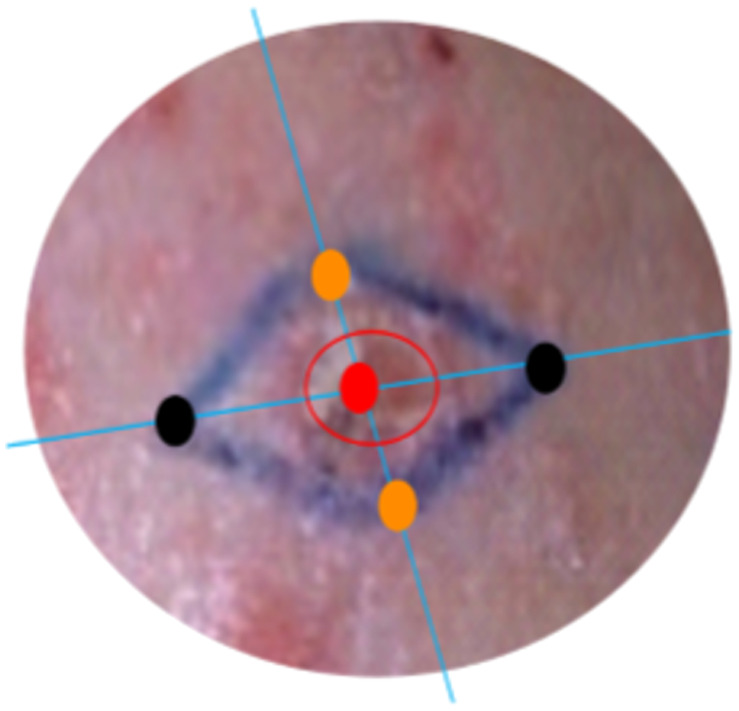
Schematic representation of the spectroscopic measurement spots on the lesion (L•), peri-lesion (PL•), and non-lesion (NL•) areas in and around the lesion area (red circle) and along the short and long axes (blue lines) of the blue ink delimited surgical spindle.

For each suspected lesion, five different locations were defined for single-point spectroscopic measurements (see Fig. 2): one at the “Lesional” area itself (L), two at a short “Peri-Lesion” distance from the lesion (PL), and two more at the edge of this safety margin area marked under the guidance of a plastic surgeon, which is supposed as “non-lesion” (NL) healthy skin. After spectroscopic acquisitions, suspicious lesions were excised along the boundaries of the marked area for the sake of a clean surgical suture.

For each patient, the acquisition protocol started with a skin surface close examination by the surgeon who delineated the three types of skin areas based on close-eye examination: a first area suspected of being a carcinoma, a second area corresponding to safety surgical margins, and a third type of area corresponding to skin evaluated as healthy based on clinical aspects and located on both edges of the surgical spindle. As ink is used to guide the surgeon during surgical resection of the spindle, all measurements were made on the three types of skin area before using ink to delineate them for surgery. Therefore, all spectroscopic measurements were made without ink on the skin. After being done with the five spectroscopic measurements (i.e., L, 2 PL, and 2 NL), the surgeon used ink to mark the spectroscopic measurement spots always according to the same protocol: L and 2 PL sites aligned together so one single histological cut could provide diagnostics for the three spots (i.e., L site in between the 2 PL sites). Each end of the surgical spindle was then cut on the fixed sample and identified according to their respective orientation on the patient (12 and 6 o’clock for instance or 3 and 9 o’clock or else), 12 o’clock being considered the direction from the center of the lesion to the patient’s vertex.

**Fig. 3:**
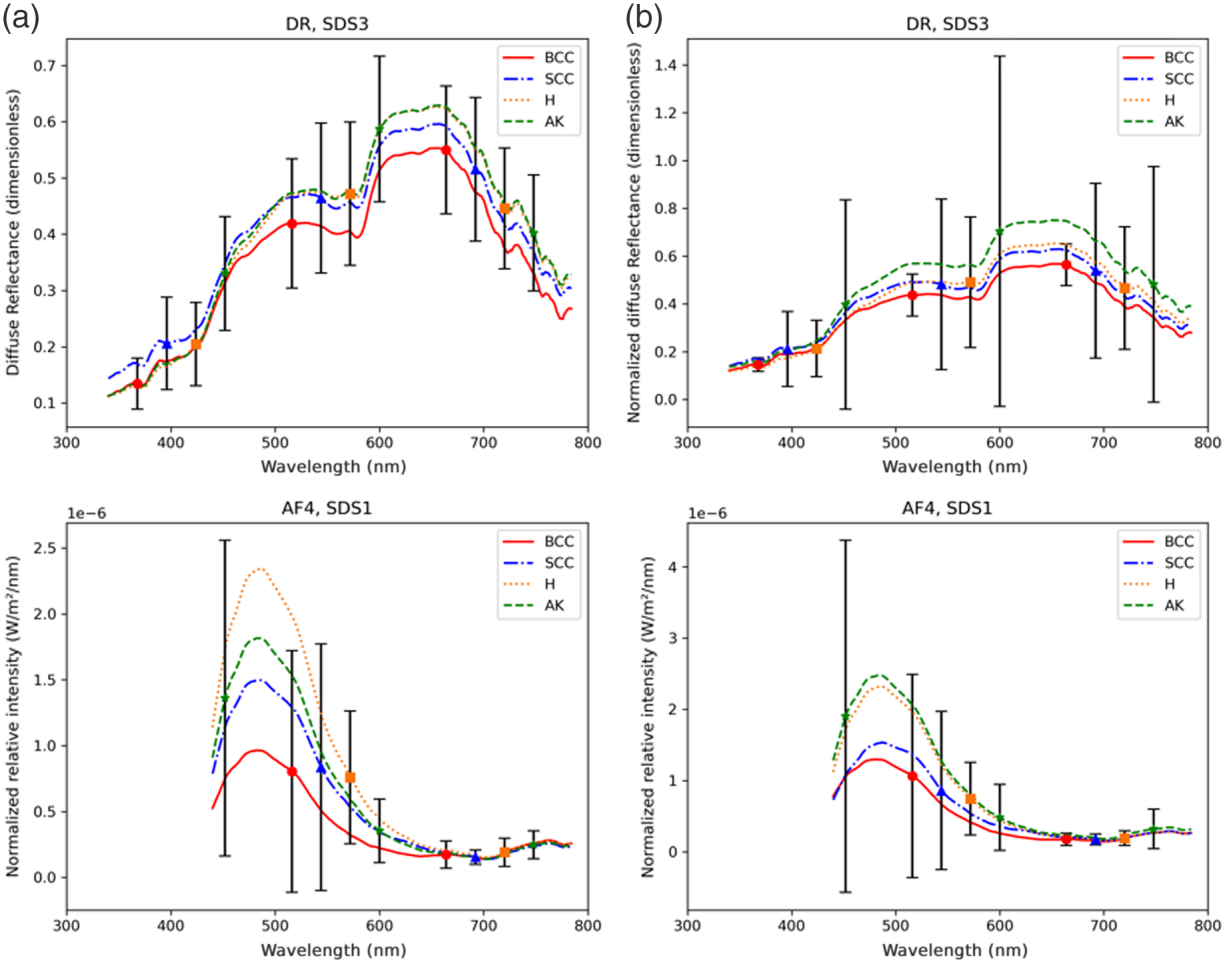
Example of average (±SD bars) DR spectra acquired at SDS3 for (a) BCC, SCC, AK, and H classes and AF spectra (λexc=395  nm) acquired at SDS1 for (b) BCC, SCC, AK, and H classes and H classes.

Based on the results of histopathological analyses acting as ground truth, each L, PL, and NL areas of the 218 anatomical sites (851 measured areas in total) were assigned to one of the four main following diagnostic classes: BCC (number of measured areas n=90), SCC (n=57), AK (n=96), or healthy skin (H, n=608). Some examples of mean spectra before and after baseline normalization for each of the four diagnostic classes, with their corresponding standard deviations (SDs), are shown in Fig. 3.

As shown in Fig. 3, for DR spectra at SDS3, before normalization, H and AK classes featured close average curves, whereas, after normalization, the DR average spectrum of the H class gets closer to the SCC DR average spectrum than to the AK one, which might be the reason why normalization did not induce any improvement in classification accuracy based on DR spectra. Regarding AF spectra, as illustrated for AF4 at SDS1 here, the baseline normalization tends to bring closer on the one hand H and AK classes and on the other hand BCC and SCC classes while enhancing the separation between H+AK and BCC+SCC groups, on average. This could explain why in the case of AF, normalization allowed an improvement in the classification accuracy values.

It can be noticed in Fig. 3 that the SD bars for each class, representing the intraclass variability in the spectral intensity curve shapes, are relatively large so that the amplitude ranges of spectra corresponding to each class overlap significantly and degrade the interclass separability. Careful optimization of every step of the data processing and classification pipeline needs to be performed to tackle this difficulty.

### 2.2 Spectroscopic Data Processing Pipeline

**Fig. 4:**
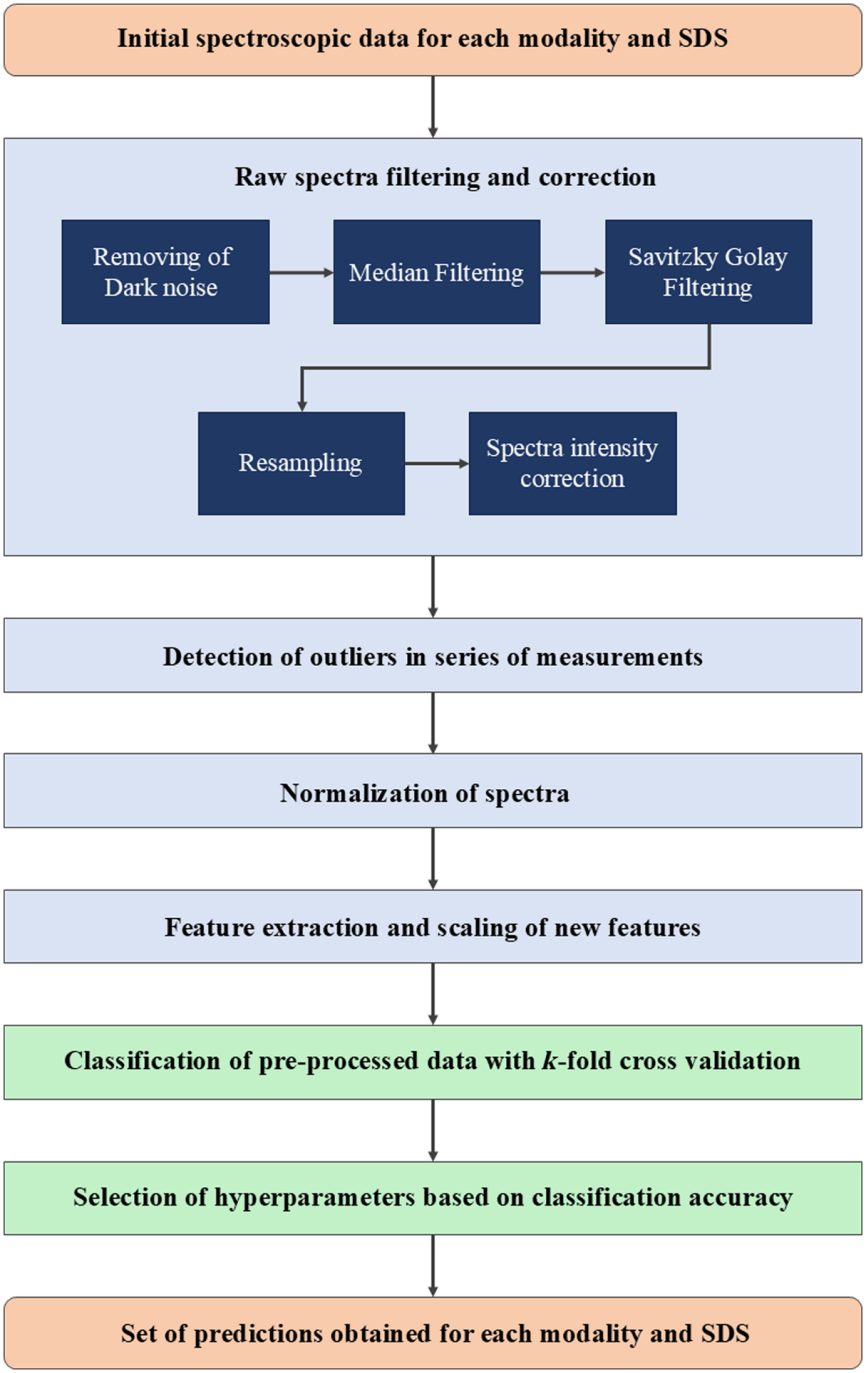
Spectroscopic data processing pipeline diagram overview.

An overview of the general scheme of the proposed data processing pipeline is given in Fig. 4. The pipeline includes: (i) raw spectra filtering and shape correction, (ii) detection of measurements that contain outliers, (iii) normalization, (iv) feature extraction, and (v) classification.

#### 2.2.1 Raw spectra filtering and correction

Pre-processing of the raw spectra of all modalities included median filtering to suppress impulse noise (window size = 3), dark noise subtraction to account for the influence of thermally generated electrons in spectrometers, smoothing using Savitzky–Golay filter (window size = 81, polynomial order = 4), and resampling (from 0.3 nm step to 1 nm step). For the AF spectra, intensity correction of the spectral response was also performed for each detection channel.

#### 2.2.2 Detection of outliers in series of measurements

As mentioned in Sec. 2.1.2, one tissue point measurement consists of three successive measurements at the same tissue point (without moving the probe). This was exploited to detect and remove any potential “outlier” signal for each series of three measurements before averaging. This outlier rejection is the same for all the modalities and SDSs and is based on relative dispersion in the form of average coefficients of variation calculated for DR and AF spectra respectively as

$${\overset{¯}{\mathrm{CV}}}_{\mathrm{AF}}=\frac{1}{N}\cdot \overset{\lambda_{2}}{\underset{\lambda_{i}\lambda_{1}}{\sum }}\frac{\sqrt{\frac{\sum_{j=1}^{3}[{\mathrm{AF}}_{j}(\lambda_{i})-\overset{¯}{\mathrm{AF}}(\lambda_{i}){]}^{2}}{3}}}{\overset{¯}{\mathrm{AF}}(\lambda_{i})},$$ (1)

$${\overset{¯}{\mathrm{CV}}}_{\mathrm{DR}}=\frac{1}{N}\cdot \overset{\lambda_{2}=780\;\mathrm{nm}}{\underset{\lambda_{i}=\lambda_{1}=340\;\mathrm{nm}}{\sum }}\frac{\sqrt{\frac{\sum_{j=1}^{3}[{\mathrm{DR}}_{j}(\lambda_{i})-\overset{¯}{\mathrm{DR}}(\lambda_{i}){]}^{2}}{3}}}{\overset{¯}{\mathrm{DR}}(\lambda_{i})},$$ (2)

where AF(λ) and DR(λ) stand for the AF and DR spectra, whereas $\overset{¯}{\mathrm{AF}}(\lambda )$ and $\bar{\mathrm{DR}}(\lambda )$ correspond to the average spectra for the three measurements of each point on the skin; $\lambda_{1}\wedge \lambda_{2}$ define the boundaries of the wavelength range used for the calculation, N is the number of points in the wavelength range of $\left[\lambda_{1};\lambda_{2}\right]$, and mean(…) and std(…) are the average and SD of the intensities of three measurements of AF or DR spectra values at each wavelength, respectively.

Coefficients of variation ${\bar{CV}}_{AF=}$ and ${\overset{¯}{CV}}_{DR=}$ are normalized as the SDs are divided by the mean of the intensity values acquired at the same wavelength. Consequently, such coefficients can be used as an independent characteristic of the “stability” (outlier presence or non-constant spectrum intensities) of a variable. On the contrary, the use of the SD without considering the mean value of the variable makes no sense because the maximum and minimum intensity values in the same spectrum can differ by order of magnitude. However, as spectra contain hundreds of points, it is more appropriate to use values averaged over the spectral range instead of exploiting the coefficient of variation for a single point.

The limits of the wavelength range for calculating ${\bar{CV}}_{AF}$ are given in Table 3 according to the excitation wavelength. Lower bounds ($\lambda_{1}$) correspond to the shortest wavelength in the transmitted band of the fluorescence emission filters, whereas higher bounds ($\lambda_{2}$) were defined as the longest wavelength beyond which a significant increase of the noise level in the spectra was observed.

**Table 3:** Wavelength ranges [$\lambda_{1}:\lambda_{2}$] nm for the calculation of the average variation coefficients of AF spectra.

Modality	$\lambda_{1}$ (nm)	$\lambda_{2}$ (nm)
${\mathrm{AF}}_{1}$, ${\mathrm{AF}}_{2}$, and ${\mathrm{AF}}_{3}$	421	550
${\mathrm{AF}}_{4}$ and ${\mathrm{AF}}_{5}$	451	580

Checking for the presence of outliers for each series of three measurements for the same skin area before averaging was performed separately for each modality and SDS and consisted of two stages. At the first stage, the mean coefficient of variation for the series of three measurements was calculated, and if its value did not exceed 0.08, then the series of measurements was considered to contain no outliers and all three spectra were used for subsequent averaging. In the opposite case, a separate coefficient of variation was calculated for each of the three possible pairs of measured spectra. The main idea of this approach was to assume that if two of the three spectra in a series of measurements were slightly different from each other and significantly different from the third, the coefficient of variation for the corresponding pair of spectra should be smaller than the coefficients of variation for the other two pairs of spectra. Thus, if one of the coefficients of variation calculated for pairs of spectra was <0.08, the corresponding pair of spectra was considered correctly measured and used for subsequent averaging. At the same time, if all coefficients of variation calculated for the three pairs of spectra exceeded 0.08, the measurement was considered incorrect and was removed from subsequent analyses for the corresponding combination of modality and SDS.

**Fig. 5:**
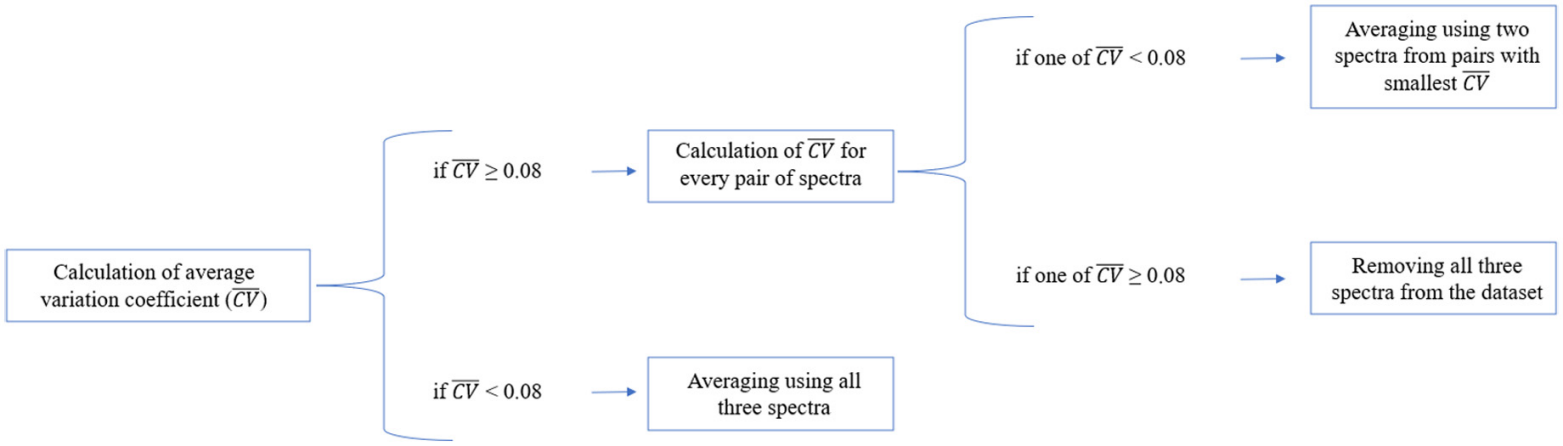
General scheme of filtering of outliers in measurements.

Indeed, the threshold value calculated on trios and pairs of spectra was determined as a tradeoff between (i) identifying and excluding measurements in which one or all three spectra were significantly different from the others and (ii) limiting the number of spectra removed from the dataset. The limit value of 0.08 was, on the one hand, small enough to filter out outliers in the measurements and, on the other hand, allowed to keep on average more than 85% of the spectra corresponding to non-healthy classes in the analyses for each modality and SDS. The general scheme of filtering outliers in measurements is shown in Fig. 5.

#### 2.2.3 Normalization of spectra

The anatomical site where spectra were taken and the age, gender, and phototype of the patients impact the shape and intensity of the measured spectral curves. Appropriate methods of spectral signal normalization have to be implemented as most of the data classification methods are sensitive to scale. In the present study, three normalization methods were compared: two intensity (maximum and ratio) and a baseline normalization method.

The first intensity normalization method consists of dividing each spectrum by its maximal intensity value lying in the spectral range of 420 to 600 nm for the AF spectra [see Eq. (3)] and in the range of 630 to 690 nm for the DR spectra [Eq. (4)]. The search range for finding the maximum for DR spectra was fixed so that the influence of hemoglobin absorption on the shape of the spectral curve is much lower than the influence of light scattering. The search range for normalization of the AF spectra was notably chosen notably to avoid the presence of a large amount of noise for all wavelengths above 600 nm

$${\mathrm{AF}}_{i}^{n}(\lambda )=\frac{{\mathrm{AF}}_{i}(\lambda )}{\max[{\mathrm{AF}}_{i}(420-600\;\mathrm{nm})]},$$ (3)

$${\mathrm{DR}}_{i}^{n}(\lambda )=\frac{{\mathrm{DR}}_{i}(\lambda )}{\max[{\mathrm{DR}}_{i}(630-690\;\mathrm{nm})]}.$$ (4)

The second intensity normalization method (ratio), proposed by Lim et al.,^34^ consists of standardizing DR and AF spectra to a baseline or reference point, as defined in Eqs. (5) and (6)

$${\mathrm{AF}}_{i}^{n}(\lambda )={\mathrm{AF}}_{i}(\lambda )\cdot \frac{\overset{¯}{{\mathrm{AF}}^{H}}}{\max[\overset{¯}{{\mathrm{AF}}_{i}^{H}}(\lambda )]},$$ (5)

$${\mathrm{DR}}_{i}^{n}(\lambda )={\mathrm{DR}}_{i}(\lambda )\cdot \frac{\overset{¯}{{\mathrm{DR}}^{H}}}{\max[\overset{¯}{{\mathrm{DR}}_{i}^{H}}(630-690\;\mathrm{nm})]},$$ (6)

where ${\mathrm{AF}}_{i}(\lambda )$ and ${\mathrm{DR}}_{i}(\lambda )$ respectively stand for the acquired AF and DR spectra of lesions or healthy skin measured near the lesion and i is the index of the lesion; $\overset{¯}{{\mathrm{AF}}^{H}}$ and $\bar{{\mathrm{DR}}^{H}}$ relate to the average intensity over the wavelength of the corresponding AF and DR spectra of healthy skin, and $\bar{{\mathrm{AF}}_{i}^{H}}(\lambda )$ and $\bar{{\mathrm{DR}}_{i}^{H}}(\lambda )$ correspond to the average AF spectra of healthy skin measured near the corresponding lesion. Here, the main idea was to normalize the intensity of the spectra using spectra of healthy skin without changing the shape of the spectral curves as ratios $\frac{\bar{{\mathrm{AF}}^{H}}}{\max[\bar{{\mathrm{AF}}_{i}^{H}}(\lambda )]}$ and $\frac{\bar{{\mathrm{DR}}^{H}}}{\max[\bar{{\mathrm{DR}}_{i}^{H}}(\lambda )]}$ are constant scalar values. The 630- to 690-nm range of the DR spectra was chosen by these authors as the wavelength interval where the contribution of light scattering to the shape of the spectral curve is mainly influenced by the hemoglobin and melanin absorption.

However, as mentioned above, anatomical site, phototype, and other parameters of the skin have a significant influence not only on the peak intensity of the spectra but also on their shape. As a consequence, the use of normalization methods affecting only the scale of the spectra may not be sufficient. Therefore, we also implemented a third normalization method with reference to the average shape of healthy spectra used as the baseline

$${\mathrm{AF}}_{i}^{n}(\lambda )={\mathrm{AF}}_{i}(\lambda )\cdot \frac{\overset{¯}{{\mathrm{AF}}^{H}}(\lambda )}{\overset{¯}{{\mathrm{AF}}_{i}^{H}}(\lambda )},$$ (7)

$${\mathrm{DR}}_{i}^{n}(\lambda )={\mathrm{DR}}_{i}(\lambda )\cdot \frac{\overset{¯}{{\mathrm{DR}}^{H}}(\lambda )}{\overset{¯}{{\mathrm{DR}}_{i}^{H}}(\lambda )},$$ (8)

where $\bar{AF_{i}^{H}}(\lambda )$ and $\bar{DR_{i}^{H}}(\lambda )$ respectively correspond to the average AF and DR spectra of healthy skin measured in the area around the respective lesion and i is the index of the lesion; $\overset{¯}{{\mathrm{AF}}^{H}}(\lambda )$ and $\bar{{\mathrm{DR}}^{H}}$ relates to the average AF and DR spectra of healthy skin for each SDS, respectively. Thus, the main difference from the method proposed by Tunnell is that the normalization ratio is not calculated on average but for each wavelength separately.

#### 2.2.4 Feature extraction

The large number of points in each spectrum can make their analysis a difficult task because working with many variables requires more computational resources and can also negatively affect the accuracy of the classification algorithm. To avoid this, the last step of spectra preprocessing was the dimensionality reduction. Two methods were tested: principal component analysis (PCA) with different kernels and autoencoder (AE) with various layer architectures and activation functions. The choice of PCA and AE was due, on the one hand, to their high performance in similar studies and, on the other hand, to the large number of customizable hyperparameters to improve the final classification accuracy. The selection of the optimal PCA kernels, AE, and activation functions as well as of the corresponding parameters (number of PCs for PCA, number of layers, and size of each layer for AE) was based on the final classification accuracy and the time required to apply the method.

#### 2.2.5 Classification model selection and evaluation

SVM, multi-layer perceptron (MLP), and linear discriminant analysis (LDA) were chosen as classifiers as all these methods showed high efficiency in solving the classification problem in studies on skin cancer diagnosis and other pathologies using spectroscopy. The best classification schemes and corresponding parameters for each group of spectra were selected based on the average classification accuracy of five iterations of the same sequence of spectra processing and classification to obtain reliable results. Given the limited and unbalanced number of individuals in each class of our data set, a stratified k-fold cross-validation (CV) strategy (k=3) was implemented to split our dataset into a training/validation set (used to tune both parameters and hyperparameters on the same set) on the one hand and a test set (used for final performance evaluation) on the other hand. K-fold CV is one of the best train-test splitting methods in the case of “small” size datasets because it ensures that all observations of the data set have a chance to appear in training and test sets, thus avoiding overtraining of the classification model due to incorrect division of the original dataset. It allows us to adjust and validate the model using the k−1 folds (training) and the remaining k’th folds (validation), respectively. The process is repeated until each k-fold has been used for training and the final performance evaluation obtained is the average of the latter scores registered. A stratified approach was implemented to maintain the percentage of samples of each class in all folds.

The value of parameter k was chosen as equal to 5 as it allowed us to obtain a test dataset of 40 or more samples for each iteration of the CV.

In all classification tests, the class sizes were equally balanced to avoid the influence of different numbers of samples per class on the final classification accuracy defined as

$$\text{Accuracy}=\frac{\sum_{1}^{N}TP_{i}}{N},$$ (9)

where ${\mathrm{TP}}_{i}$ corresponds to the number of correctly predicted samples for lesional classes (i=SCC, BCC, CI, CNI, or AK) or healthy skin (i=H) and N relates to the number of classes.

Before each series of calculations, samples were randomly selected for all classes except the one for which the number of samples was the smallest. The selection of the best-performing classifier and corresponding hyperparameters was based on the average final classification accuracy for all CV tests. A list of the methods used and the corresponding hyperparameters are presented in Table 4. The parameter variation ranges were chosen based on the results of a previous study.^56^ The entire sequence, starting from random sample selection was repeated five times to obtain statistically reliable results.

**Table 4:** Data analysis methods and optimized hyperparameters.

	Method	Optimized parameters
Feature extraction	Principal component analysis	Kernel, number of components
	Autoencoder	Number of layers, size of each layer, type of solver
Classification	SVM	Kernel, decision function, regularization parameter value
	MLP	Number of layers, size of each layer, type of solver
	LDA	—

Training and optimization of the classification models took a few dozen seconds/a few minutes (PC Intel®Core i7-11850H, RAM 32Gb, Windows 11) depending on whether the method has a small/large number of tunable hyperparameters.

#### 2.2.6 Binary- and multi-class classifications

To investigate the ability of optical spectroscopy to discriminate skin lesions according to their histological type and features, one set of binary and multi-class classifications was performed between carcinomas (BCC and SCC), precancer (AK), and healthy (H) classes, and another set between CI and CNI, precancer (AK), and healthy (H) classes. Invasiveness is indeed one of the features that have a direct impact on surgery in terms of surgical safety margin lengths.^58^ This way, we can evaluate the interest that optical spectroscopy may have for dermatologists and surgeons in terms of surgical guidance. Furthermore, a global cancer class (KC) was also considered for comparisons when grouping BCC and SCC (or CI and CNI), whereas AK and H classes were kept separated. The results are presented both in a binary mode and a multi-class mode to better assess the clinical relevance of using the method (combining optical spectroscopy and ML classification) in a real clinical environment. In this case, the method will be used by a healthcare professional who will have previously sorted the skin lesions and needs diagnostic orientation assistance to confirm or not his diagnostic visual assessment among the different possible classes most common in the case of carcinomatous lesions: H, AK, SCC, and BCC.

### 2.3 Data Fusion

The last stage of spectra processing was the fusion of decisions for each sample using all the predictions obtained for each modality and each SDS. Fusion was performed using three different methods: majority voting (MV), voting with weighted voting for each modality and SDS (MO) and stacking (S), which is the use of an ML classifier to make a diagnosis based on the predictions obtained after the classification step.

**Fig. 6:**
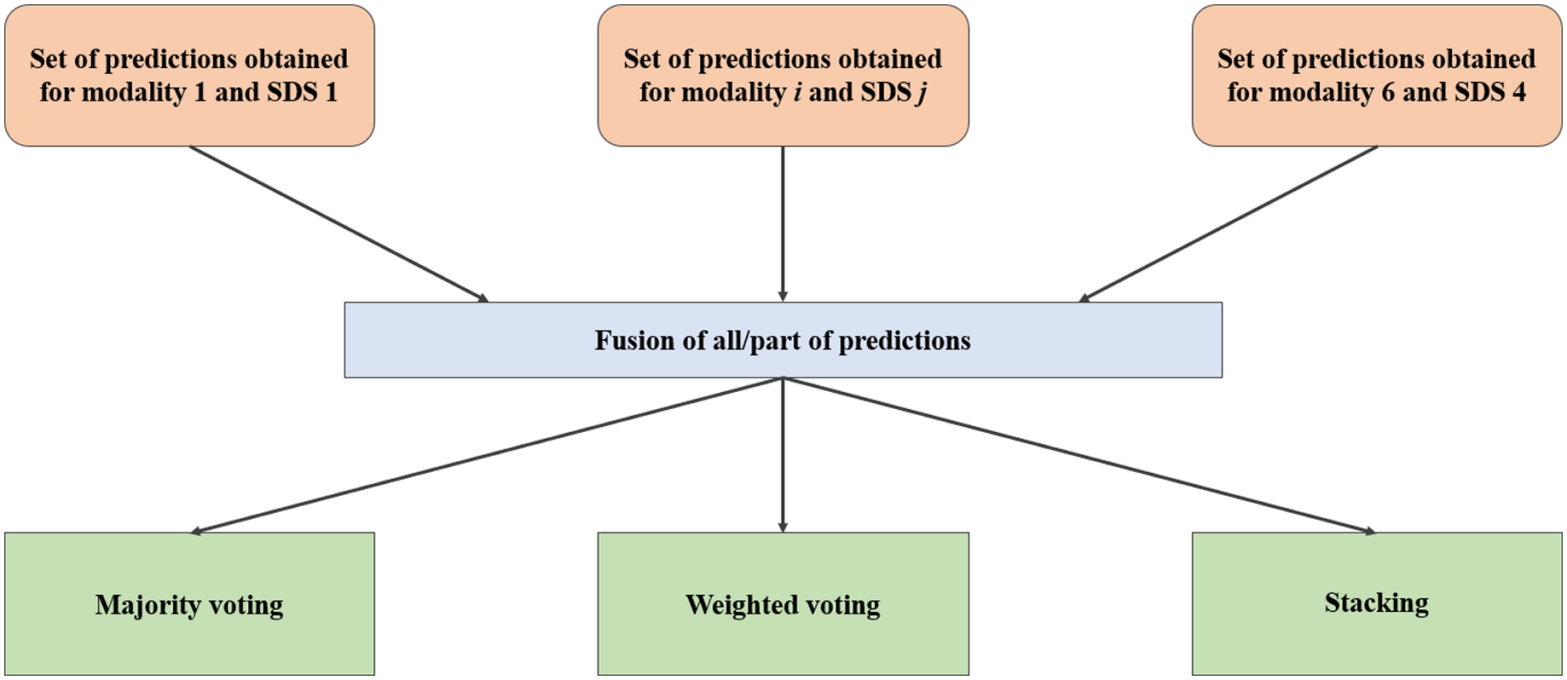
General scheme of fusion of decisions.

The grouping of the data for fusion was done at three different levels: fusion of all results, fusion of the results obtained for each modality, and fusion of the results obtained for each SDS. Thus, the general scheme of data fusion is shown in Fig. 6.

## 3 Results and Discussion

### 3.1 Best Methods and Hyperparameters Selected

The selected hyperparameter values (or range of values) used in each step of the spectroscopic data classification pipeline are provided in Table 5. They correspond to the highest accuracy values calculated after binary- and multi-class classification for each combination of histological classes, spectroscopy modality, and SDS separately. As normalization has a significant effect on the performance of different data processing methods, it was also necessary to find, for each unique combination of modality, SDS, and histological classes pair, the normalization method that would provide the highest classification accuracy.

**Table 5:** Best hyperparameter values retained for each method in the spectroscopic data classification pipeline.

Data processing pipeline step	Method	Hyperparameters	
		Type	Value
Raw spectra filtering and correction	Removing dark noise	—	—
	Median filter	Kernel size	3
	Savitzky–Golay filter	Window size	∼5 nm
		Polynomial order	4
Normalization	No normalization	—	—
	Intensity (max)	—	—
	Intensity (ratio)	—	—
	Baseline	—	—
Feature extraction	Principal component analysis	Number of PCs	15 to 20
	Autoencoder	Hidden layer number	3
		Hidden layer size	80, 40, 80
Classification	SVM	Kernel	Linear, polynomial (2 to 3), radial-based function
		Decision function shape	One versus rest
	MLP	Hidden layer number	1
		Layer size	80 to 100
	LDA	—	—
Decision Fusion	MV	—	—
	Stacking	Type of classifier	SVM, random forest (80 trees)
	Weighted voting	—	—

### 3.2 Binary Classifications

#### 3.2.1 BCC, SCC, AK, and H histological classes

**Fig. 7:**
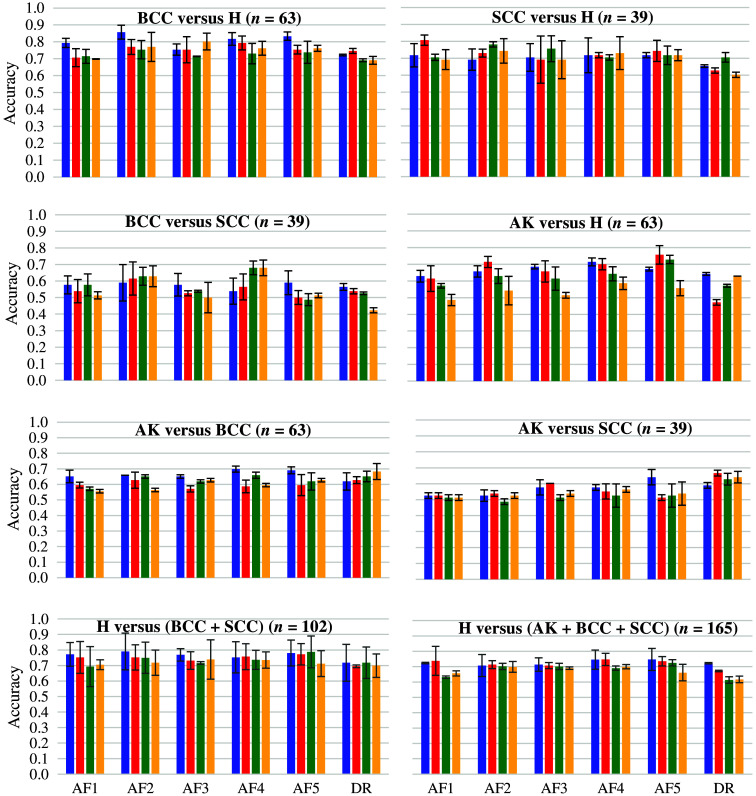
Mean classification accuracy of pairwise binary classifications obtained for every spectroscopic modality (autofluorescence AF1.5, diffuse reflectance DR) and source-to-detector separation (${\mathrm{SDS}}_{1}$ in blue, ${\mathrm{SDS}}_{2}$ in red, ${\mathrm{SDS}}_{3}$ in green, and ${\mathrm{SDS}}_{4}$ in orange) among the four histological classes: BCC, SCC, AK (actinic keratoses), and H (healthy skin) classes. The error bars indicate the SDs obtained after five independent repeats of the whole sequence of spectra processing.

**Fig. 8:**
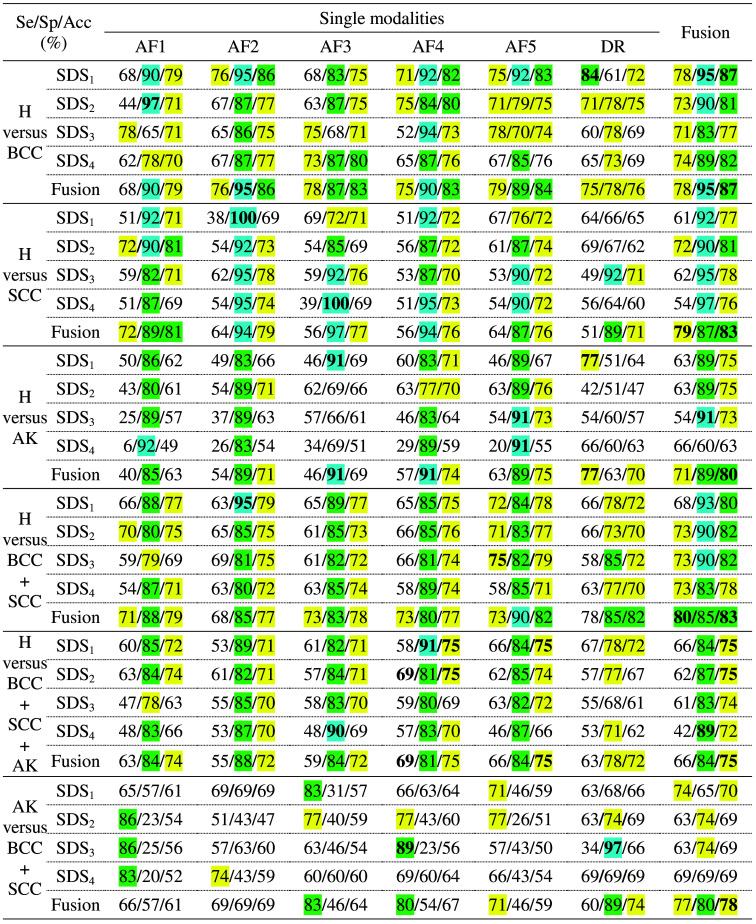
Classification sensitivity (Se), specificity (Sp), and accuracy (Acc) obtained for BCC, SCC, AK, and H (healthy) classes pairwise discrimination for every spectroscopic modality (autofluorescence AF1.5, diffuse reflectance DR) and source to detector separation (${\mathrm{SDS}}_{1\ldots 4}$) and their fusions. Values are highlighted in colors blue, green, and yellow, which correspond to ≥90%, ≥80%, and ≥70%, respectively. The best values are in bold.

The accuracy results of pairwise binary classifications between histological classes BCC, SCC, AK, and H, obtained for combinations of each spectroscopic modality alone (five AF excitations and DR) and SDS (from 400 to 1000  μm), are presented in Fig. 7. Figure 8 shows the results of binary classification using our ML pipeline, including data fusion. In this figure, sensitivity (Se)/specificity (Sp)/accuracy (Acc) values in % are given for every single modality (in columns, five AF excitation + DR), for every SDS (in rows), and for their fusions. It is to be noticed that (i) best results for all pairs of classes were obtained using baseline normalization for AF spectra, no normalization for DR spectra, PCA and SVM classifier with a linear kernel for each modality, (ii) the effectiveness of decision fusion methods varies for different pairs of classes, and (iii) weighted voting and stacking performed better than MV.

The highest classification accuracies in single modalities (above 81%) were obtained when discriminating H class from each cancerous class: H versus BCC (86% based on the combination of ${\mathrm{AF}}_{2}$ and ${\mathrm{SDS}}_{1}$), H versus SCC (81% using ${\mathrm{AF}}_{1}$ and ${\mathrm{SDS}}_{2}$). For H versus BCC, the highest Se of 84% was obtained for DR and the shortest for SDS while Sp exceeded 92% for AF1, AF2, and AF5 at short ${\mathrm{SDS}}_{1}$ and ${\mathrm{SDS}}_{2}$. The fusion of all modalities and SDS finally provided the highest Acc (87%) with improved Se (78%) and high Sp (95%). For H versus SCC, the values of Sp and Acc are slightly lower compared with BCC, but the fusion step allowed the improvement of Se and Acc by 7 and 2 percentage points (pp) respectively compared with single modalities and SDS while maintaining high Sp of 95%. When comparing H class to all KC (BCC + SCC), we observed that the overall single modality accuracy decreased (79% in the best case) compared with H versus BCC or to H versus SCC. After applying decision fusion, the classification accuracy increased to 83% for H versus KC, similar to the H versus SCC case. The Se value is also further increased when using a fusion strategy to reach 80% while maintaining a high Sp value of 85%. Although the classification accuracy was one of the lowest for discriminating BCC from SCC (between 55% and 70% for all SDS and light source combinations), it is of interest to mention that the ability to differentiate between two such carcinoma classes is of interest, especially in the case of patients follow up as BCC grows more slowly than SCC.^59^

The decrease in classification accuracy when considering H versus BCC (87%) to the situation of discriminating H versus BCC+SCC (83%) is due to the drop in specificity, which is high for H versus BCC (95%) and lower for H versus SCC (87%) and H versus BCC+SCC (83%). This drop in specificity may be explained by the variable spectroscopic pattern featured by the two histological classes (Fig. 3). Such variable patterns may confuse the classification pipeline compared with the situation when each cancerous class is taken apart from each other. Indeed, most BCC and SCC feature typical histological patterns such as tumor retractation spaces for BCC^60^ or keratin mass for SCC.^61^ Such morphologic differences may impact how light interacts with each type of KC: the variability of interactions between light and skin tissues increases the variability of spectroscopic features of another class.

When comparing H versus AK, we observed that DR at ${\mathrm{SDS}}_{1}$ provided the highest Se value (77%), whereas AF3 at ${\mathrm{SDS}}_{1}$ and AF5 at long SDS provided the highest Sp value (91%). Here again, fusion improved the Acc by 4% to reach 80% with good Sp (89%) but moderate Se (71%) compared for instance with single modality DR and ${\mathrm{SDS}}_{1}$ (77%). The corresponding low/high values of true positive/negative highlight the difficulty/ability of the model to correctly classify AK/H tissues, respectively.

For H versus rest (BCC, SCC, and AK together), high Sp of 91% (AF4 and ${\mathrm{SDS}}_{1}$) was obtained with limited Acc (75%) due to a low Se of 58%, meaning that, in this case, also a high number of false negative was found (more cases of lesions are missed). Although specificity values were significantly higher (10 to 40 pp) than sensitivity values in almost all single modality cases, here again, the fusion of decision provided the most balanced classification model among all three criteria (Se = 66%, Sp = 84%, and Acc = 75%).

Finally, comparing AK class versus KC (BCC + SCC), we can see that the highest accuracy value (78%) was obtained after fusion (+9 pp compared with the highest Acc for single modality), whereas the highest Se/Sp values (89%/97%) were obtained for single modalities AF4/DR at ${\mathrm{SDS}}_{3}$, respectively. When discriminating AK from other histological classes, accuracy values did not exceed 70%: 67% for AK versus SCC ($\mathrm{DR}\text{-}{\mathrm{SDS}}_{2}$ and ${\mathrm{SDS}}_{3}$), 69% for AK versus H (${\mathrm{AF}}_{5}\text{-}{\mathrm{SDS}}_{1}$), and 70% for AK versus BCC (${\mathrm{AF}}_{4}\text{-}{\mathrm{SDS}}_{1}$). The same level of accuracy was obtained when discriminating cancerous classes from one another: 68% for SCC versus BCC (${\mathrm{AF}}_{4}\text{-}{\mathrm{SDS}}_{4}$). The decrease in classification accuracy when AK (72%) is included compared with the classification situation not including AK (80%) highlights the fact that AK is particularly difficult to discriminate both from H and from cancerous lesions from a spectroscopic point of view. Indeed, AK is an “intermediate” histological class described in the literature as a precancerous stage displaying common histological features both with healthy skin and SCC.^62^ Therefore, the lowest classification accuracy is obtained, both before and after decision fusion, when discriminating AK and SCC, which can be explained by the fact that stage 3 AKs may be considered *in situ* SCC: hence, depending on the pathologist’s habits, AK are sometimes classified either in SCC or in AK. The training set of data may therefore be confusing for the classifier.

Globally, classification accuracy achieved the best results after decision fusion when the AK histological class was not included among the classes to discriminate: classification accuracy is between 75% and 80% when AK is included, whereas it is above 83% when AK is not included. The highest accuracy values were obtained when discriminating H from BCC, SCC, or cancer (BCC+SCC). Furthermore, in most cases, decision fusion allowed us to obtain a good balance among Se, Sp, and Acc values, which highlights the diagnostic interest of combining AF and DR spectroscopic modalities compared with the single modality approach. Pioneering studies in our scientific field have presented classification accuracy results obtained on datasets, including 18 BCCs, 4 SCCs, and 6 AK,^35^ or on 28 BCCs and SCCs^36^ or on 19 BCCs, 38 SCCs and 14 Aks,^34^ compared with the current study (63 BCC, 39 SCC, and 63 AK). These three studies present binary classification results in terms of specificity of 71% (BCC versus H), or diagnostic accuracy of 99% (BCC+SCC versus H) and 87% (BCC+SCC+AK versus H). Only one study presents a result (90%-accuracy) discriminating a cancerous class versus a precancerous class: BCC+SCC versus AK. Our results (87% for BCC versus H and 82% for BCC+SCC versus H) are comparable to those already published.

It is clear from Figs. 7 and 8 that for all pairs of classes not including AK, the classification accuracy obtained using DR spectra single modality is slightly lower at all distances than for one or several AF modalities, which may be related to the greater influence of carcinogenesis on the concentrations of endogenous skin fluorophores, as a consequence of which the separation of cancerous and healthy skin using AF spectra becomes easier. At the same time, the changes in various optical parameters during AK development are smaller than during carcinogenesis as it is a precancerous condition. As a consequence, the features that contain AF spectra become less suitable for the separation of diagnostic classes, making the classification accuracy of AF spectra reduced to the level of classification accuracy of DR spectra.

**Fig. 9:**
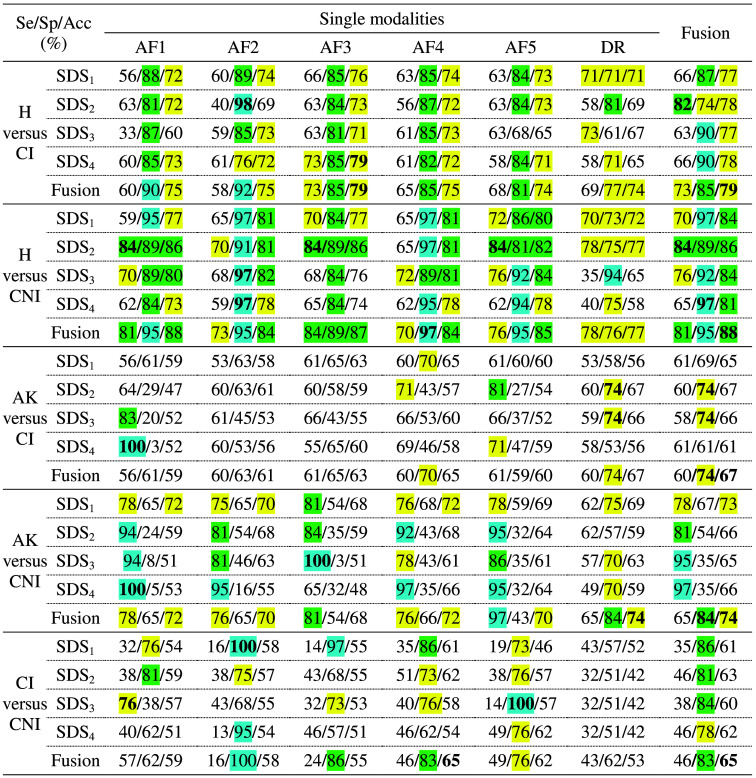
Classification sensitivity (Se), specificity (Sp), and accuracy (Acc) obtained for CI (invasive carcinoma), CNI (noninvasive carcinoma), AK, and H (healthy) classes pairwise discrimination for every spectroscopic modality (autofluorescence AF1.5, diffuse reflectance DR) and source to detector separation (${\mathrm{SDS}}_{1\ldots 4}$), and their fusions. Values are highlighted in colors blue, green, and yellow, which represent ≥90%, ≥80%, and ≥70%, respectively. The best values are in bold.

As shown in Figs. 8 and 9, the best classification rates were obtained for all pairs of classes to be discriminated by fusing decisions from all light sources and all SDSs. If we exclude the classification results obtained following data fusion and analyze the results obtained by each light source and each SDS separately, ${\mathrm{SDS}}_{1}$ and ${\mathrm{SD}}_{2}$ gave the best results for all pairs of classes to be discriminated. Light acquired at short distances tends to travel in upper layers, i.e., the epidermis where cancer initially develops. This might explain why classification results tend to be better for data acquired at short distances than for longer ones. However, the fusion of decisions from the four SDS always increased the classification results compared with a single SDS considered separately, which highlights the interest in using a device featuring several SDSs such as the one used in the current study.

#### 3.2.2 CI, CNI, AK, and H classes

**Fig. 10:**
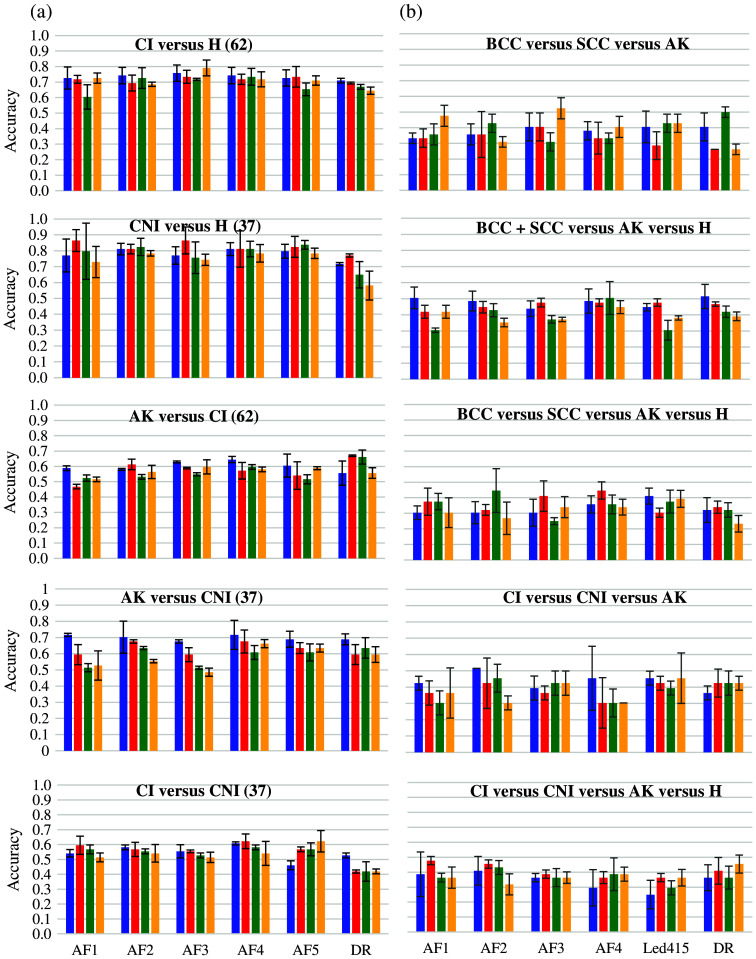
Mean classification accuracy of pairwise binary-class (a) and multi-class (b) classifications obtained for every spectroscopic modality (autofluorescence AF1.5, diffuse reflectance DR) and source to detector separation (${\mathrm{SDS}}_{1}$ in blue, ${\mathrm{SDS}}_{2}$ in red, ${\mathrm{SDS}}_{3}$ in green, and ${\mathrm{SDS}}_{4}$ in orange) among the four histological classes: CNI (non-invasive carcinoma), CI (invasive carcinoma), AK (actinic keratoses), and H (healthy skin) classes. The error bars indicate the SDs obtained after five independent repeats of the whole sequence of spectra processing.

The accuracy results of pairwise binary classifications between histological classes CI, CNI, AK, and H, obtained for combinations of each spectroscopic modality alone (five AF excitations and DR) and SDS (from 400 to 1000  μm), are presented in Fig. 10(a). Figure 9 shows the results of binary classification using our ML pipeline including data fusion, with Se, Sp, and Acc values given for every single modality (in columns, five AF excitation and DR), for every SDS (in lines), and for their fusions.

We observe +9pp in Acc for H versus CNI (+8pp/+10pp for Se/Sp) compared with H versus CI, with a major contribution of AFs regardless of the SDS. For CI versus CNI, AK is better discriminated from CNI (+10pp) than from CI. For AK versus CI, AF1 with long SDS 3 to 4 and DR provided the best Se and Sp, respectively. For AK versus CNI, AFs with SDS2-4 performed better in Se and DR better in Sp. For, CI versus CNI, Se was globally low and AF2-5 performed best for Sp. Overall, in all cases, the fusion brings the highest Acc with the most balanced Se/Sp couples.

Based on single modalities, a higher classification accuracy was obtained for H versus CNI (86% for ${\mathrm{AF}1\text{-}\mathrm{SDS}}_{2}$ and ${\mathrm{AF}3\text{-}\mathrm{SDS}}_{2}$) than for H versus CI (79% for ${\mathrm{AF}3\text{-}\mathrm{SDS}}_{4}$). As for BCC and SCC classes, discriminating CI and CNI classes from one another or AK from cancerous classes induced decreased classification accuracies: 62% for CNI versus CI (AF4,5 and ${\mathrm{SDS}}_{2,4}$), 67% for AK versus CI ($\mathrm{DR}\text{-}{\mathrm{SDS}}_{2}$), and 72% for AK versus CNI (${\mathrm{AF}}_{1}\text{-}{\mathrm{SDS}}_{1}$ and ${\mathrm{AF}}_{4}\text{-}{\mathrm{SDS}}_{1}$). The use of decision fusion improved classification accuracy by 2 or 3 pp for the discrimination of CNI class from H (88%), AK (74%), and CI (65%).

The lower classification accuracy obtained when discriminating H from CI (77%) than from CNI (86%) may be explained by the fact that BCCs included in the current study were mostly non-invasive. Therefore, the CI class was composed of 51% of BCC (49% of SCC), and the CNI class was composed of 78% of BCC. As the classifier reaches higher accuracy when discriminating H from BCC than from SCC, the CNI class was better discriminated from H than CI from H as CI includes a greater number of SCC that are more difficult for the classifier to discriminate from H (see Sec. 3.2.1).

When considering invasiveness as the main histological criteria to define classes, shorter wavelengths (${\mathrm{AF}}_{1}$: 365 nm and ${\mathrm{AF}}_{3}$: 395 nm) tended to provide better classification accuracies than longer wavelengths that provided the best results for discrimination of healthy skin compared with conventional histological classes (BCC, SCC). Invasiveness is associated with matrix metallopeptidase expression^63^ by cancerous cells and therefore with collagen degradation of the dermis. As collagen excitation maximum wavelength is 350 nm, ${\mathrm{AF}}_{1}$ (365 nm) is more sensitive than longer excitation wavelengths (${\mathrm{AF}}_{5}$ in our case) to collagen fluorescence emission modification due to dermal collagen degradation.

The decreasing accuracy rates of the CNI class from H (88%), AK (74%), and CI (65%) were expected as CNI does not display histological features that are common with healthy skin (H). However, non-invasive carcinomas (CNI) share common histological features with CI as there are BCC and SCC included both in CNI and CI. Again, AK being an intermediate class between H and cancerous states (CI and CNI), the classification accuracy is also itself intermediate.

When comparing the results obtained for different modalities, patterns similar to those in the previous paragraph are observed: Sp is significantly higher than Se in most cases, and the classification accuracy values of the DRS are slightly lower for cases where there is no AK present in the pairs of classes.

As described by Maher et al. in their study on inter-rater concordance of BCC subtypes,^64^ infiltrating BCC was one of the subtypes for which inter-rater concordance (among seven raters) was 0.49 according to Light’s Kappa statistics which considers that one is the perfect agreement. A Light’s Kappa score below 0.6 is considered a moderate agreement. This result should be kept in mind when interpreting the classification accuracy of optical spectra in the current study as a degree of uncertainty may also be considered for the reference histological classification. As an example, a sample classified as a non-invasive BCC by the pathologist (although displaying minor invasive features) may be classified as invasive by spectroscopy.

### 3.3 Multiclass Classification

The use of a binary classification may not be sufficient to prove the clinical relevance of the method. Taking this fact into account, the results of multiclass classifications are presented in Fig. 10(b) and Table 6 in the form of confusion matrices, which provide the percentage of the actual class (in rows) corresponding to the predicted class (in columns). Moreover, the use of weighted voting-based decision fusion improved classification accuracy by 5 to 10 pp compared with the best single modality classifications.

**Table 6:** Confusion matrices obtained after decision fusion for multiclass classification for: three classifications: (a) H versus AK versus (BCC + SCC), (b) AK versus BCC versus SCC, and (c) AK versus CNI versus CI, and four-class classification: (d) H versus AK versus BCC versus SCC and (e) H versus K versus CNI versus CI, with BCC, SCC, AK (actinic keratoses) and H (healthy skin). Best correct classification rates are highlighted in bold; worst incorrect classification rates are highlighted in italics.

Total accuracy = 59%				
%	BCC+SCC	H	AK	
(a)				
BCC+SCC	**69**	14	17	
H	14	**72**	14	
AK	*29*	*42*	29	
Total accuracy = 54%				
%	BCC	SCC	AK	
(b)				
BCC	**64**	7	29	
SCC	*43*	*43*	14	
AK	43	0	**57**	
Total accuracy = 57%				
%	CI	CNI	AK	
(c)				
CI	*27*	*27*	46	
CNI	0	54	46	
AK	0	9	**90**	
Total accuracy = 50%				
%	BCC	SCC	H	AK
(d)				
BCC	29	21	*36*	14
SCC	29	42	*29*	0
H	0	14	**79**	7
AK	7	14	29	**50**
Total accuracy = 57%				
%	CI	CNI	H	AK
(e)				
CI	**46**	9	*36*	9
CNI	46	*18*	*27*	9
H	0	0	**100**	0
K	0	18	18	**63**

To start with the first three class situation (Table 6a), considering H, AK, and cancers gathering BCC and SCC, 72% of H were well classified, whereas the rest was misclassified as cancer or AK; and 69% of all cancers were well classified, whereas the rest was misclassified as H or AK. These results are in good agreement with the corresponding bi-class results in terms of Se and Sp values (see Sec. 3.2.1). Furthermore, we can notice a small proportion of H and cancers (14% and 17%, respectively) were misclassified as AK, whereas samples labeled as AK class were mostly classified as cancers or H and only 29% as AK. Indeed, this result highlights the difficulty of classifying AK depending on their more or less benign or malignant state. In Table 6b, comparing the three classes of lesions between them (AK, BCC, and SCC), we observe acceptable precision values for BCC and AK but a higher difficulty in correctly classifying SCC-labelled lesions, of which 43% were classified as BCC and 14% as AK. In Table 6c, AK is highly discriminated (90%) from CI and CNI, whereas CNI is confused with AK in 46% of cases. CI appears to be the most difficult class to classify and spread among CI, CNI, and AK.

To continue with the four-class situations (Table 6d), consistent with results in Table 6a, the precision value for healthy skin is quite high (79%), whereas the precision values for all pathologies do not exceed 50%. Half of AK samples are mainly classified as H and as SCC, which can be explained by the intermediate character of this histological “precancerous” class. Regarding BCC and SCC, a significant number of samples were misclassified as healthy (false negative), which poses a problem of sensitivity of the method. In Table 6e, this misclassification is confirmed for CI and CNI versus H; however, in this case, it is to be noticed that all H samples are well classified as well as most AK leading to a total 57% accuracy.

Argenziano et al.^65^ found that, after a 1-day training course in KC diagnostics, 88 general practitioners (2522 patients) achieved a 54%-diagnostic accuracy for BCC. As shown in Table 6, decision fusion achieved the same level of diagnostic accuracy up to 57% for three-class tests (SCC+BCC versus AK versus H) and 57% for a four-class test CNI versus CI versus AK versus H. Such results are very encouraging to consider evaluating the interest in bimodal optical spectroscopy in areas featuring a low dermatological workforce. It was estimated that four dermatologists per 100,000 population are needed to adequately care for a community. According to Glazer and Rigel,^66^ 59% of the USA territory displays three dermatologists or less for 100,000 inhabitants. Some areas even display no dermatologists, which may imply a late diagnosis. Optical spectroscopic systems could provide diagnostic assistance to pharmacists or nurses, who outnumber doctors in such areas, and thus could represent a solution for a first level of referral either in an emergency to a specialist if necessary or for regular monitoring. Although building the classification model may take up to 10 min (usually <2  min), once model training and optimization are completed, the classification of every new measured spectrum using the trained model takes only a few seconds. A software engineer is currently developing within our team a unique software integrating the data acquisition program (equipment control), the pre-processing program (noise removal, filtering, optical correction, etc.), and the classification program, which is the subject of this paper. Initial tests have been carried out that confirm the possibility of displaying the diagnostic classification result of a new measurement point on the skin in “real-time,” i.e., in a few seconds.

## 4 Conclusion and Prospects

The current study provides diagnostic accuracies that are comparable to the ones achieved in previous studies also based on the combination of several spectroscopic modalities and machine-learning methods: multi-class classification diagnostic accuracies were obtained on four classes for the first time, at levels comparable to the classification accuracies featured by general practitioners thanks to decision fusion. Such encouraging results imply the device development to its next step. Indeed, the current study presents results obtained by data processing performed offline: data were acquired using dedicated software, then stored in a database, pre-processed, and finally used to develop classification models. The next step in the development of the spectroscopic device will be to implement models online to provide real-time diagnostic aid: new data will be acquired and compared with the developed models. Finally, a classification result will be provided. Next, a clinical study will be dedicated to the evaluation of the reliability of these models in terms of positive and negative predictive values. New data will also expand the set of spectra used to train the diagnostic models, which should have a positive impact on classification accuracies. In the next steps of this project, a multicentric clinical trial is foreseen, which will allow for the collection of supplementary clinical and bimodal spectroscopic data. Considering a larger dataset (i.e., including more individuals in each diagnostic class), deep learning techniques should therefore be investigated as a possible solution to further optimize our data analysis methodology. Based on the results obtained in the future, the number of light sources and spectrometers (i.e., measurement distances SDS from the excitation central optical fiber) may be reduced and in case the spectroscopic data processing solution may provide high accuracy with a reduced number of information sources.

## Data Availability

The data presented in this article are publicly available in their raw format along with metadata and pre-processing program at https://doi.org/10.12763/EYVX3P. Given that an industrial transfer of the device (hardware and software) is envisaged, the code will not be put online on a public repository but can be made (partly) available from the corresponding author upon request.
